# Chemical Structures and Biological Activities of Limonoids from the Genus *Swietenia* (Meliaceae)

**DOI:** 10.3390/molecules23071588

**Published:** 2018-06-29

**Authors:** Yun-Peng Sun, Wen-Fang Jin, Yong-Yue Wang, Gang Wang, Susan L. Morris-Natschke, Jin-Song Liu, Guo-Kai Wang, Kuo-Hsiung Lee

**Affiliations:** 1 School of Pharmacy, Anhui University of Chinese Medicine, Hefei 230012, China; syp1good@163.com (Y.-P.S.); jwfmiji@163.com (W.-F.J.); wyy_1996@126.com (Y.-Y.W.); kunhong_8@163.com (G.W.); jinsongliu@ahtcm.edu.cn (J.-S.L.); 2 Synergetic Innovation Center of Anhui Authentic Chinese Medicine Quality Improvement, Hefei 230012, China; 3 Natural Products Research Laboratories, Eshelman School of Pharmacy, University of North Carolina, Chapel Hill, NC 27599-7568, USA; susan_natschke@unc.edu; 4 Chinese Medicine Research and Development Center, China Medical University and Hospital, Taichung 40402, Taiwan

**Keywords:** genus *Swietenia*, limonoids, chemical components, biological activities

## Abstract

*Swietenia* is a genus in the plant family Meliaceae. This genus contains seven to eight known species, found in the tropical and subtropical regions of the Americas and West Africa. Thus far, more than 160 limonoids have been isolated from four species of the genus *Swietenia*. Limonoids are rich in structure type and biological activity, and these compounds are the main active components in the *Swietenia* species. This paper will give a comprehensive overview of the recent phytochemical and pharmacological research on the terpenes from *Swietenia* plants and encourage further drug discovery research.

## 1. Introduction

The genus *Swietenia* (Meliaceae) includes 7–8 species, which are mainly distributed in the tropical and subtropical regions of the Americas. These plants have gradually been introduced and cultivated in Indonesia, Vietnam, and Yunnan, Gansu, and other regions of China, and have grown well. The species *Swietenia mahagoni* J. acq, *Swietenia macrophylla* King and *Swietenia humilis* Zucc are timber species widely used in traditional medicine. In addition, *Swietenia aubrevilleana*, a hybrid of *S. mahagoni* and *S. macrophylla*, has been increasingly studied [1]. Prior reports have validated some of the traditional uses or found promising bioactivities in the laboratory, such as antidiabetic [2,3,4], antimicrobial [5,6,7], antioxidant [8,9], anti-inflammatory [10], antitumor [11,12], and acaricidal properties [13]. Previous phytochemical studies revealed that various types of limonoids have been isolated from *Swietenia* species, along with smaller amounts of steroids, coumarins, phytosterols [14], lignans [15], polyphenols [16], and essential oils [17] also found.

Limonoids, classified as tetranortriterpenoids, are formed by the loss of four terminal carbons from a side chain on an apotirucallane or apoeuphane skeleton with cyclization to form a 17β-furan ring [18]. Limonoids are found mainly as important secondary metabolites in the fruits of Rutaceae and Meliaceae plants. Their extensive biological effects, such as anti-malarial [19], antifeedant [20], insecticidal [21], and antitumor properties [22], have attracted the attention of many researchers. Structurally, most limonoids found in the genus *Swietenia* are classified as mexicanolide- and phragmalin-type, but the structural types are abundant and varied.

## 2. Chemical Components

### 2.1. Azadirone-Type and Evodulon-Type Limonoids

**Figure 1:**
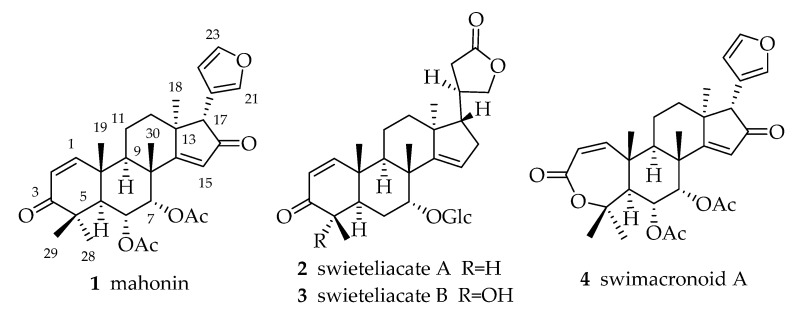
Chemical structures of azadirone-type and evodulon-type limonoids **1**–**4**.

Azadirone-type limonoids are characterized by a 3-oxo-Δ^1,2^ pattern and C-7 oxygenation. Mahonin (**1**) was first isolated from the cotyledons of *S. mahagoni* in 1989 [23] and republished by the same author in 1990 [24,25]. The structures of swieteliacates A and B (**2** and **3**), which contain a lactone ring rather than the more common furan ring at C-17, were first reported in 2018 [26]. Swimacronoid A (**4**), an evodulon-type limonoid, was obtained from *S. macrophylla* in 2013 [27] (Figure 1).

### 2.2. Gedunin-Type Limonoids

**Figure 2:**
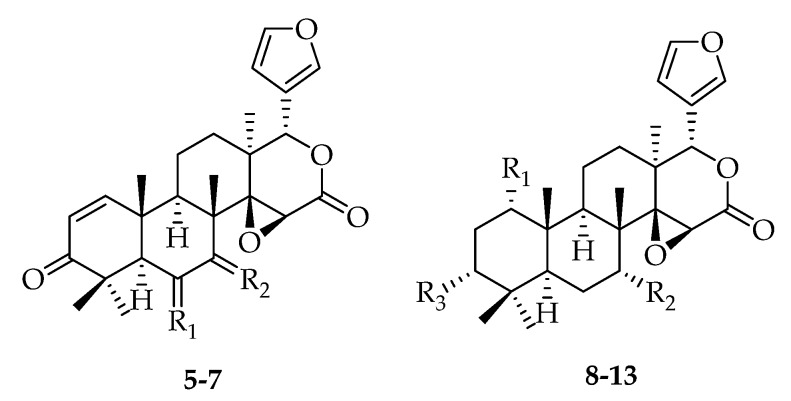
Chemical structures of gedunin-type limonoids **5**–**13**.

Gedunin-type limonoids with a δ-lactone in ring D are derived from the azadirone class via a Baeyer-Villiger type ring expansion. 7-Deacetoxy-7-oxogedunin (**5**) was obtained from *S. mahagoni*, together with 6*α*-acetoxygedunin (**6**) [24]. Compound **5** was also isolated from *S. macrophylla* [28] and *S. aubrevilleana* [1]. In 2009, compounds **8**–**13** were isolated from the fruits of *S. mahagoni* [29] (Figure 2, Table 1). 

### 2.3. Andirobin-Type Limonoids

**Figure 3:**
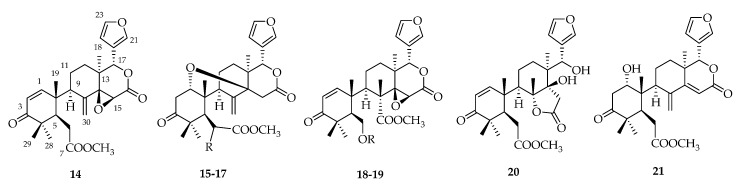
Chemical structures of andirobin-type limonoids **14**–**21**.

Andirobin-type limonoids are characterized by cleavages between C-7/8 and C-16/17 as well as the formation of a Δ8,30 exocyclic double bond and δ-lactone D ring. Secomahoganin (**18**) was first isolated from *S. mahagoni* in 1989 [23,24], and later from *S. macrophylla* in 2015 [33]. Multiple new andirobin-class limonoids, including deacetylsecomahoganin (**19**) [30], swiemahogin A (**20**) [34], and swietmanin J (**21**) [29], were obtained from *S. macrophylla* (Figure 3, Table 2).

### 2.4. Mexicanolide-Type Limonoids

**Figure 4:**
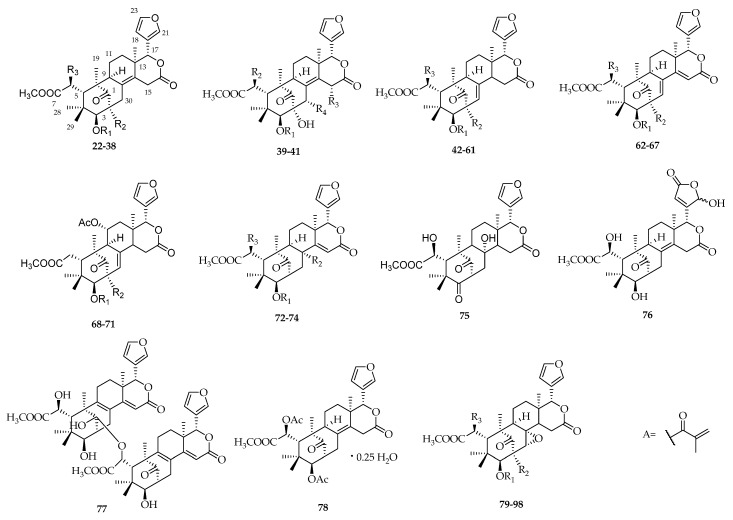
Chemical structures of mexicanolide-type limonoids **22**–**98**.

A total of 77 mexicanolide-type limonoids, **22**–**98**, have been reported from *Swietenia* species, and most were isolated from *S. mahagoni* and *S. macrophylla.* In a few reports, mexicanolide-type limonoids have also been found in *S. humilis*, for example, humilin B (**88**) [38], humilinolides A–H (**89**–**90**, **50**–**52**, **91**, **61**, **98**) [39,40,41,42], and 2-hydroxy-destigloyl-6-deoxyswietenine acetate (**60**) [42]. Swietenolide (**23**), 6-*O*-acetylswietenolide (**25**), and 3,6-*O*,*O*-diacetylswietenolide (**27**) were also obtained from *S. aubrevilleana* [1]. Other related interesting structures have been found in *Swietenia* species. Kadota et al. discovered a novel dimeric limonoid, mahagonin (**77**), from an oily fraction of the ether extract of *S. mahagoni* [43]. In addition, compound **78** was extracted from the seeds of *S. macrophylla*. The crystal structure contains 0.25 molecules of water and is stabilized by O–H···O and weak C–H···O hydrogen bonds [44] (Figure 4, Table 3).

### 2.5. Phragmalin-Type Limonoids

**Figure 5:**
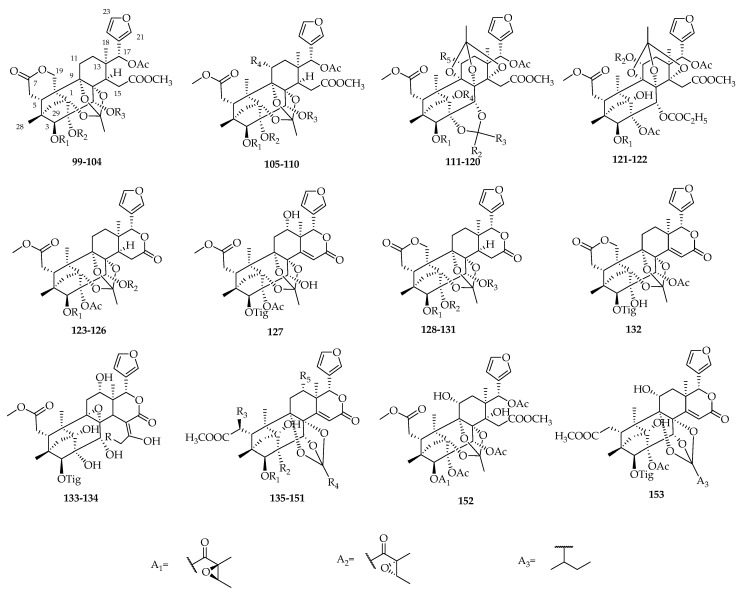
Chemical structures of phragmalin-type limonoids **99**–**153**.

Totally, 55 phragmalin-type limonoids, **99**–**153**, have been reported from *S. mahagoni* and *S. macrophylla.* Among them, 27 novel phragmalin-type limonoids, swietenitins A–X (**99**–**103**, **106**–**107**, **114**–**116**, **121**–**125**, **127**–**134**), 2,11-diacetoxyswietenialide D (**108**), 11-deoxyswietenialide D (**109**), 2-acetoxyswietenialide D (**110**), together with a known compound, epoxyfebrinin B (**126**), were published successively in 2009 [63] and 2011 [64]. Moreover, swietephragmins A–F (**135**–**141**) were obtained from *S. mahagoni* [30] and swietephragmins H–J (**149**–**151**) were found in *S. macrophylla* [65]. In 2008, compounds **142**–**147** were isolated from *S. macrophylla* [66]*.* Thereafter, compounds **148** and **153** with similar structures have been reported [14,31] (Figure 5, Table 4).

### 2.6. Polyoxyphragmalin-Type Limonoids

**Figure 6:**
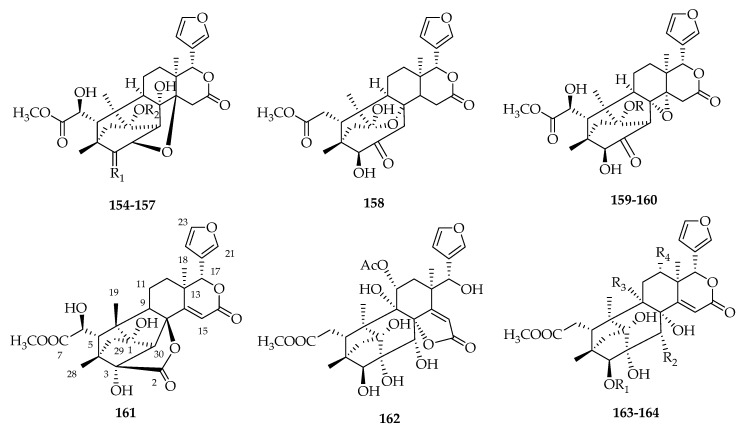
Chemical structures of polyoxyphragmalin-type limonoids **154**–**164**.

Currently, only 11 polyoxyphragmalin-type limonoids have been isolated from *Swietenia* species. Among them, seven known compounds, khayanolide E (**154**), 1-*O*-acetylkhayanolide B (**155**), 1-*O*-deacetylkhayanolide E (**156**), khayanolide B (**157**), khayalactone (**158**), 1-*O*-acetylkhayanolide A (**159**) and khayanolide A (**160**), were isolated from *S. macrophylla* [37]. The structure of swietemahalactone (**161**), an example of a novel rearranged polyoxyphragmalin-type limonoid, was confirmed by X-ray crystallographic analysis [67]. Similarly, a rearrangement of the lactone ring occurred in the structure of **162** [34]. Compounds **163** and **164** were discovered from *S. macrophylla* in 2012 and 2009 (Figure 6, Table 5).

## 3. Biological Activities

### 3.1. Antifeedant Activity

Table 6 lists the 50% antifeedant index concentration (DC_50_), minimum antifeedant concentration (MAC), and antifeedant index (AI, mean ± SEM) values of the antifeedant activity in studies using Meliaceous limonoids and *Spodoptera* insects. At 20 μg/leaf-cm^2^ (1000 ppm), swietemahonin G (**85**) strongly inhibited the larval feeding of *Spodoptera littoralis* and swietephragmins **135**–**141** showed moderate activity [30]. Swietenialides A–E (**111**–**113**, **117**, **118**) showed antifeedant activity at 1000 ppm concentration against the third-instar larvae of *S. littoralis* (Boisduval) [36]. Swietenolide (**23**), 6-*O*-acetylswietenolide (**25**), 3,6-*O*,*O*-diacetylswietenolide (**27**), swietenine (**42**), 2-hydroxyswietenine (**55**) and swietemahonin F (**84**) were evaluated at concentrations of 1000 ppm against the final instar larvae of *Spodoptera frugiperda* [1]. Among these five limonoids from *S. macrophylla* and *S. aubrevilleana,* swietenine (**42**) showed the greatest potency with a DC_50_ value of 2.49 ± 1.44 (mg/L). These limonoids also inhibit larval growth inhibition activity against *Helicoverpa zea*, *Heliothis virescens* and *Manduca sexta* insect species [68].

### 3.2. Antimicrobial Activity

Eleven limonoids from *Swietenia* species were tested for antifungal activity against the groundnut rust *Puccinia arachidis*. Activity was calculated as the percent reduction in the numbers of rust pustules on treated groundnut leaflets compared with untreated control leaflets. Among these compounds, 6-acetylswietenine (**48**), 6-acetyl-3-tigloylswietenolide (**26**), 2,3-dihydroxy-3-deoxy-mexicanolide (**37**), 3*β*-hydroxymexicanolide (**30**), 3*β*-acetoxymexicanolide (**33**) and mexicanolide (**22**) showed the highest activity, causing 80–95% reduction at 10 µg/cm^2^ leaflet area, while 3,6-*O*,*O*-diacetylswietenolide (**27**) and swietenolide (**23**) exhibited moderate activity, causing over 60% reduction at the same concentration. Surprisingly, swietenine (**42**) increased the disease severity considerably at lower concentrations relative to control [46]. The antifungal effects of ten limonoids were determined by a radial growth technique. At a concentration of 1500 mg/L, deacetoxy-7-oxogedunin (**5**) inhibited *Botrytis cinerea* growth by 60.8%. This value was comparable with those found with swietenine (**42**) at 1000 mg/L (57.5%) and 3-*O*-acetylswietenolide (**24**) at 1500 mg/L (63.1%) [70]. 2-Hydroxy-3-*O*-tigloylswietenolide (**31**) and swietenolide (**23**) were tested against eight multiple-drug-resistant bacterial strains using the conventional agar disc diffusion assay. The former compound exhibited more potent antimicrobial activity than the latter compound against all tested fungi (Group A *β haemolytic Streptococcus aureus*, *Staphylococcus aureus*, *Streptococcus pneumoniae*, *Haemophilus influenzae*, *Escherichia coli*, *Klebsiella pneumoniae*, *Salmonella typhi*, and *Salmonella paratyphi*) Vancomycin (10 µg/disc) was used as the positive control. [47]. Thirty limonoids from *S. mahagoni* were inactive in antimicrobial testing against 11 microbes (seven bacteria and four fungi) in vitro. However, 2-hydroxy-3-*O*-isobutyrylproceranolide (**34**) and 2-hydroxyfissinolide (**36**) exhibited activity against *Micrococcus luteus* ATCC 9341 with MIC values of 50 and 12.5 μg/mL, respectively, in a broth dilution test. Ofloxacin was used as the positive control [29].

### 3.3. Hypoglycemic Activity

When assayed for effects on peripheral glucose utilization employing an isolated rat hemidiaphragm method, swietenine (**42**) exhibited significant (*p* < 0.01) activity comparable with that of human insulin (*p* < 0.01) [71]. In the same year, the same compound was also found to exhibit significant dose-dependent hypoglycemic and hypolipidemic activity in type 2 diabetic rats when given by oral administration at 25 and 50 mg/kg body weight per day [72]. Dewanjee et al. obtained similar conclusions in 2011 [73]. Compounds **60**, **54** and **88** were active (3.16–31.6 mg/kg, bw) when tested as hypoglycemic agents in normal and NA–STZ-hyperglycemic mice [42]. Three *S. macrophylla* bioactive compounds, 6-*O*-acetylswietenolide (**25**), 3,6-*O*,*O*-diacetylswietenolide (**27**), and swietenine (**42**), induced uptake of glucose by muscle cells by increasing the translocation of GLUT4 to the plasma membrane. The limonoids exhibited a good potential for anti-diabetic activity, however, with a minimal side effect of weight gain [33].

### 3.4. Anti-PAF Activity

Kadota et al. published the first example of limonoids having antagonistic effects on PAF, finding the following rank order of inhibition at 100 μg/mL: swietemahonin A (**79**), 97.4%; swietemahonin E (**83**), 91.7%; 3-*O*-acetylswietenolide (**24**), 91.6%; swietenolide (**23**), 35.2% [52]. In other examples, swietemahonins A, D, E, G (**79**, **81**–**82**, **85**), 3-*O*-acetylswietenolide (**24**) and 6-*O*-acetylswietenolide (**25**), strongly inhibited PAF-induced aggregation of rabbit platelets in vitro, giving IC_50_ values of 40.2, 40.3, 51.2, 42.6, 52.9, 80.4 and 55.6 μg/mL. The same study reported that swietemahonin E (**83**) reduced PAF-induced mortality in mice [51].

### 3.5. Anti-Inflammatory Activities

*6-O*-Acetyl-3′-demethylswietephragmin E (**148**), 3,6-*O*,*O*-diacetylswietenolide (**27**), 3-*O*-tigloyl-swietenolide (**28**), 3-*O*-tigloyl-6-*O*-acetylswietenolide (**26**), swietemahonin E (**83**), methyl 3*β*-tigloyloxy-2-hydroxy-8*α*,30*α*-epoxy-l-oxomeliacate (**95**), and 6-*O*-acetylswietemahonin G (**96**) inhibited formyl-l-methionyl-l-leucyl-l-phenylalanine (fMLP)-induced superoxide anion generation with IC_50_ values of 27.6–48.7 μM. The assay was based on the superoxide dismutaste (SOD)-inhibitable reduction of ferricytochrome *c* and used ibuprofen as the positive control. Among all tested compounds, **96** was the most potent against O${}_{2}^{\text{·}-}$ generation. A 8α, 30α-epoxy group was beneficial, and acetyl substitution at C-6 was preferable to hydroxy or no substitution [14]. In addition, swietemacrophin (**97**) and humilinolide F (**91**) exhibited moderate activity with IC_50_ values of 45.44 and 27.13 μg/mL [56].

### 3.6. Other Activities

Limonoids **7**, 1**5**, **23**, **48**, and **92** were tested for their in vitro half-maximal effective concentration against dengue virus 2 and showed inhibitory activity in the concentration range of 3.5 to 12.5 μM. Among the five limonoids, **92** exhibited significant antiviral activity (EC_50_ = 7.2 ± 1.33 μM) with a selectivity index (CC_50_/EC_50_) value greater than 27.7 [35]. Swieteliacate B (**3**) was moderately active against HL-60 and SW-480 with IC_50_ values of 30.59 and 32.68 μM [26]. 7-Deacetoxy-7-oxogedunin (**5**) was cytotoxic toward Hep-G2 cells with an IC_50_ value of 16.17 μM [74]. Humilinolides A–D (**89**–**90**, **50**–**51**) showed weak cytotoxic activity against three human tumor cell lines (A-549, MCF-7 and HT-29), and generally produced high mortality rates against larvae of *Ostrinia nubilalis* [40]. Similarly, when tested against the growth of *O*. *nubilalis*, humilinolide E (**52**) and methyl-2-hydroxy-3β-isobutyroxy-1-oxomeliac-8(30)-enate (**53**) showed comparable effects to those of the positive control, toosendandin, in terms of reduction of % pupation and % adult emergence, while humilin B (**88**) and swietenine C were effective only for adult emergence [41]. Five limonoids, swietenolide (**23**), 3,6-*O*,*O*-diacetylswietenolide (**27**), swietenine (**42**), swietemahonin G (**85**), and 2-hydroxyswietenine (**55**), isolated from *S. macrophylla* and *S. aubrevilleana*, were tested in the *Artemia salina* lethality assay. Only **85** showed weak activity (LC_50_ 220.1 ppm); however, certain semi-synthetic structural modifications led to increased toxicity. The addition of acyl groups, particularly benzoyl groups, was quite effective; for example, 6-*O*-benzoylswietenolide (LC_50_ 4.3 ppm) and 6-*O*-benzoylswietenine (LC_50_ 7.5 ppm) were significantly more active than the non-acylated parent compounds **23** and **42**, respectively (LC_50_ > 500 ppm) [75]. Humilinolide A (**89**) can cause intestinal spasmogenic and uterotonic action [61]. Swietephragmin H (**149**) and swietephragmin I (**150**) possessed low anti-oxidative effects (17.12 ± 0.49% and 13.43 ± 0.28%, respectively) at the highest concentration (320 μg/mL) tested. These two compounds lack H-atom donating ability and electron delocalised potential, which are important structural features for significant antioxidant potency [65]. Local injection of mexicanolide (**22**) (0.5–3.5 mg) led to concentration-dependent antihyperalgesic action in NA-STZ hyperglycemic mice [76].

## 4. Conclusions

**Figure 7:**
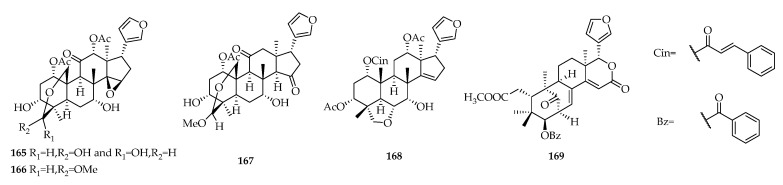
Chemical structures of limonoids **165**–**169** from other plants.

*Swietenia* is a genus in the subfamily mahogany (Meliaceae), which is generally considered to contain 7 to 8 species. Among them, the seeds and bark of *S. mahagoni*, *S. macrophylla* and *S. humilis* are used in folk medicines for the treatment of hypertension, diabetes, malaria, and epilepsy in Indonesia, India and Mexico [2,76,77]. Based on the data available, this paper summarizes five types of limonoids and describes various bioactive activities, such as antifeedant, hypoglycemic, antimicrobial, anti-PAF, anti-inflammatory, antitumor, insecticidal, anti-oxidative and antihyperalgesic. Although most of the limonoids isolated from *Swietenia* species do not show significant antiproliferative effects against cancer cell lines, some structurally similar limonoids isolated from *Melia azedarach* exhibit good antitumor activity. The best known compound is toosendanin (**165**), which strong inhibits multiple tumor cell lines; its IC_50_ values were 0.005, 0.009 and 0.0054 μM against HL60, AZ521 and U937, respectively [78,79]. In addition, meliarachin C (**166**), 12-dehydro-29-exo-neoazedarachin D (**167**), and 1-*O*-cinnamoyltrichilinin (**168**) exhibited IC_50_ values ranging from 0.65 to 9.1 μM against HL60 [79]. Erythrocarpine A (**169**), isolated from *Chisocheton erythrocarpus,* showed cytotoxicity against P388 murine leukemia cells with IC_50_ value of 2.0 μg/mL [80] (Figure 7). Its structure differs from that of seenganolide A (**67**) only by the presence of a benzoyl ester rather than hydroxy group. Therefore, limonoids from the genus *Swietenia* still have great potential for biological activity and may be modified structurally to improve their activity.

Furthermore, the published research on *Swietenia* has been focused mostly on the seeds and their limonoid components; however, but other plant parts and other compound types may also have rich pharmacological activities. Therefore, it is extremely urgent to expand the scope of research on *Swietenia* and discover or develop additional biologically active constituents of this plant genus.

## Figures and Tables

**Table 1:** Structures and sources of gedunin-type limonoids **5**–**13**.

No.	Compounds	Substitution Groups	Sources
**5**	7-deacetoxy-7-oxogedunin	R_1_ = H_2_, R_2_ = O	*S. mahagoni* [24,29,30,31] *S. macrophylla* [1,28,32], *S. aubrevilleana* [1]
**6**	6*α*-acetoxygedunin	R_1_ = R_2_ = *β-*H, *α-*OAc	*S. mahagoni* [24],
**7**	7-deacetoxy-7*α*-hydroxygedunin(deacetylgedunin)	R_1_ = H_2_, R_2_ = *β*-H, *α*-OH	*S. macrophylla* [28], *S. aubrevilleana* [1]
**8**	3-deacetylkhivorin	R_1_ = OAc, R_2_ = OAc, R_3_ = OH	*S. mahagoni* [29]
**9**	3,7-dideacetylkhivorin	R_1_ = OAc, R_2_ = OH, R_3_ = OH	*S. mahagoni* [29]
**10**	1,3,7-trideacetylkhivorin	R_1_ = OH, R_2_ = OH, R_3_ = OH	*S. mahagoni* [29]
**11**	khivorin	R_1_ = OAc, R_2_ = OAc, R_3_ = OAc	*S. mahagoni* [29]
**12**	7-deacetylkhivorin	R_1_ = OAc, R_2_ = OH, R_3_ = OAc	*S. mahagoni* [29]
**13**	1-deacetylkhivorin	R_1_ = OH, R_2_ = OAc, R_3_ = OAc	*S. mahagoni* [29]

**Table 2:** Structures and sources of andirobin-type limonoids **14**–**21**.

No.	Compound	Substitution Groups	Sources
**14**	andirobin		*S. macrophylla* [1,35]
**15**	methylangolensate	R = H	*S. mahagoni* [24,29,31,36], *S. macrophylla* [27]
**16**	6-hydroxy derivative (methyl 6-hydroxyangolensate)	R = OH	*S. mahagoni* [29,30,36,37], *S. aubrevilleana* [1], *S. macrophylla* [27]
**17**	6-acetoxyangolensate	R = OAc	*S. macrophylla* [27]
**18**	secomahoganin	R = Ac	*S. mahagoni* [23,24,25], *S. macrophylla* [33]
**19**	deacetylsecomahoganin	R = H	*S. mahagoni* [30], *S. macrophylla* [27]
**20**	swiemahogin A		*S. mahagoni* [34]
**21**	swietmanin J		*S. mahagoni* [29]

**Table 3:** Structures and sources of mexicanolide-type limonoids **22**–**98**.

No.	Compounds	Substitution Groups	Sources
**22**	mexicanolide	R_1_ = O, R_2_ = H, R_3_ = H	*S. mahagoni* [29]
**23**	swietenolide	R_1_ = H, R_2_ = H, R_3_ = OH	*S. mahagoni* [24,45,46,47], *S. aubrevilleana* [1], *S. macrophylla* [1,32,48,49,50]
**24**	3-*O*-acetylswietenolide	R_1_ = Ac, R_2_ = H, R_3_ = OH	*S. mahagoni* [24,46,51,52], *S. macrophylla* [32,48]
**25**	6-*O*-acetylswietenolide	R_1_ = H, R_2_ = H, R_3_ = OAc	*S. mahagoni* [24,51], *S. macrophylla* [1,48,53], *S. aubrevilleana* [1]
**26**	3-*O*-tigloyl-6-*O*-acetylswietenolide	R_1_ = Tig, R_2_ = H, R_3_ = OAc	*S. mahagoni* [24,46], *S. macrophylla* [14,32,48],
**27**	3,6-*O*,*O*-diacetylswietenolide	R_1_ = Ac, R_2_ = H, R_3_ = OAc	*S. mahagoni* [24,46,51], *S. macrophylla* [1,14,48,50,54], *S. aubrevilleana* [1]
**28**	3-*O*-tigloylswietenolide	R_1_ = Tig, R_2_ = H, R_3_ = OH	*S. mahagoni* [24,46], *S. macrophylla* [14,48,55],
**29**	khayasin T	R_1_ = Tig, R_2_ = H, R_3_ = H	*S. mahagoni* [24,29], *S. macrophylla* [1,14,48]
**30**	proceranolide	R_1_ = H, R_2_ = H, R_3_ = H	*S. mahagoni* [24,48], *S. macrophylla* [32,33]
**31**	2-hydroxy-3-*O*-tigloylswietenolide	R_1_ = Tig, R_2_ = OH, R_3_ = OH	*S. mahagoni* [30,47]
**32**	3-*O*-propionylproceranolide	R_1_ = COEt, R_2_ = H, R_3_ = H	*S. macrophylla* [48]
**33**	fissinolide	R_1_ = Ac, R_2_ = H, R_3_ = H	*S. macrophylla* [32,33,48], *S. mahagoni* [29]
**34**	2-hydroxy-3-*O*-isobutyrylproceranolide	R_1_ = iBu, R_2_ = OH, R_3_ = H	*S. mahagoni* [29]
**35**	2-hydroxy-3-*O*-benzoylproceranolide	R_1_ = Bz, R_2_ = OH, R_3_ = H	*S. mahagoni* [29]
**36**	2-hydroxyfissinolide	R_1_ = Ac, R_2_ = OH, R_3_ = H	*S. mahagoni* [29]
**37**	2,3-dihydroxy-3-deoxymexicanolide	R_1_ = H, R_2_ = OH, R_3_ = H	*S. mahagoni* [29]
**38**	2-hydroxy-6-deoxyswietenolide tiglate	R_1_ = Tig, R_2_ = OH, R_3_ = H	*S. mahagoni* [29]
**39**	augustineolide	R_1_ = Tig, R_2_ = OH, R_3_ = OAc, R_4_ = OiBu	*S. macrophylla* [1]
**40**	swietmanin E	R_1_ = Tig, R_2_ = H, R_3_ = OH, R_4_ = H	*S. mahagoni* [29]
**41**	swietmanin F	R_1_ = Ac, R_2_ = H, R_3_ = OH, R_4_ = H	*S. mahagoni* [29]
**42**	swietenine	R_1_ = Tig, R_2_ = H, R_3_ = OH	*S. mahagoni* [24,35,45,46], *S. macrophylla* [14,33,36,48,49,56,57]
**43**	swietenine B	R_1_ = COEt, R_2_ = H, R_3_ = OH	*S. mahagoni* [24]
**44**	swietenine C	R_1_ = iBu, R_2_ = H, R_3_ = OH	*S. mahagoni* [24,58], *S. humilis* [41]
**45**	swietenine D	R_1_ = A, R_2_ = H, R_3_ = OH	*S. mahagoni* [24]
**46**	swietenine E	R_1_ = Piv, R_2_ = H, R_3_ = OH	*S. mahagoni* [24]
**47**	swietenine F	R_1_ = Bz, R_2_ = H, R_3_ = OH	*S. mahagoni* [24]
**48**	swietenine acetate (6-*O*-acetylswietenine)	R_1_ = Tig, R_2_ = H, R_3_ = OAc	*S. mahagoni* [24,46], *S. macrophylla* [14,33,49]
**49**	6-desoxyswietenine (febrifugin)	R_1_ = Tig, R_2_ = H, R_3_ = H	*S. mahagoni* [46,59], *S. macrophylla* [1,14,48]
**50**	humilinolide C	R_1_ = Tig, R_2_ = OAc, R_3_ = H	*S. humilis* [39,40,41]
**51**	humilinolide D	R_1_ = Ac, R_2_ = OH, R_3_ = OAc	*S. humilis* [39,40,41]
**52**	humilinolide E (6-*O*-acetyl-2-hydroxyswietenin)	R_1_ = Tig, R_2_ = OH, R_3_ = OAc	*S. humilis* [41], *S. mahagoni* [31,57]
**53**	methyl-2-hydroxy-3-b-isobutyroxy- 1-oxomeliac-8(30)-enate	R_1_ = iBu, R_2_ = OH, R_3_ = H	*S. humilis* [38,41]
**54**	methyl-2-hydroxy-3-b-tigloyloxy- 1-oxomeliac-8(30)-enate	R_1_ = Tig, R_2_ = OH, R_3_ = H	*S. humilis* [41], *S. macrophylla* [58], *S. mahagoni* [31]
**55**	2-hydroxyswietenine	R_1_ = Tig, R_2_ = OH, R_3_ = OH	*S. mahagoni* [31,36,56], *S. macrophylla* [1,58]
**56**	6-acetoxyhumilinolide C	R_1_ = Tig, R_2_ = OAc, R_3_ = OAc	*S. aubrevilleana* [1]
**57**	granatumin H	R_1_ = iBu, R_2_ = H, R_3_ = H	*S. macrophylla* [48]
**58**	swieteliacate C	R_1_ = COEt, R_2_ = H, R_3_ = H	*S. macrophylla* [26]
**59**	6-*O*-acetylswietenin B	R_1_ = COEt, R_2_ = H, R_3_ = OAc	*S. macrophylla* [48]
**60**	2-hydroxy-destigloyl-6-deoxyswietenine acetate	R_1_ = Ac, R_2_ = OH, R_3_ = H	*S. humilis* [42]
**61**	humilinolide G	R_1_ = iBu, R_2_ = OAc, R_3_ = H	*S. humilis* [42]
**62**	swielimonoid A	R_1_ = Tig, R_2_ = H, R_3_ = OH	*S. macrophylla* [60]
**63**	swielimonoid B	R_1_ = COEt, R_2_ = H, R_3_ = OH	*S. macrophylla* [60]
**64**	swietmanin G	R_1_ = iBu, R_2_ = OH, R_3_ = H	*S. mahagoni* [29]
**65**	swietmanin H	R_1_ = Ac, R_2_ = OH, R_3_ = H	*S. mahagoni* [29]
**66**	swietmanin I	R_1_ = Tig, R_2_ = OH, R_3_ = H	*S. mahagoni* [29]
**67**	seneganolide A	R_1_ = H, R_2_ = H, R_3_ = H	*S. mahagoni* [29]
**68**	swietmanin A	R_1_ = iBu, R_2_ = H	*S. mahagoni* [29]
**69**	swietmanin B	R_1_ = Ac, R_2_ = H	*S. mahagoni* [29]
**70**	swietmanin C	R_1_ = H, R_2_ = H	*S. mahagoni* [29]
**71**	swietmanin D	R_1_ = Ac, R_2_ = OAc	*S. mahagoni* [29]
**72**	8*α*-hydroxycarapin	R_1_ = O, R_2_ = OH, R_3_ = H	*S. mahagoni* [29]
**73**	3*β*,6-dihydroxydihydrocarapin	R_1_ = H, R_2_ = H, R_3_ = OH	*S. macrophylla* [1], *S. aubrevilleana* [1]
**74**	swieteliacate E	R_1_ = H, R_2_ = OH, R_3_ = OH	*S. macrophylla* [26]
**75**	khayanone		*S. macrophylla* [37]
**76**	swieteliacate D		*S. macrophylla* [26]
**77**	mahagonin		*S. mahagoni* [43], *S. macrophylla* [26]
**78**	3,6-di-*O*-acetylswietenolide 0.25-hydrate		*S. macrophylla* [44]
**79**	swietemahonin A	R_1_ = COEt, R_2_ = H, R_3_ = OH	*S. mahagoni* [24,45,51,52]
**80**	swietemahonin B	R_1_ = COEt, R_2_ = H, R_3_ = OAc	*S. mahagoni* [24,45], *S. macrophylla* [48]
**81**	swietemahonin C	R_1_ = iBu, R_2_ = H, R_3_ = OAc	*S. mahagoni* [24,41,45]
**82**	swietemahonin D	R_1_ = Ac, R_2_ = H, R_3_ = OH	*S. mahagoni* [24,45,51]
**83**	swietemahonin E	R_1_ = Tig, R_2_ = H, R_3_ = OH	*S. mahagoni* [24,45,51,52], *S. macrophylla* [1,14,33,48]
**84**	swietemahonin F	R_1_ = Tig, R_2_ = H, R_3_ = OAc	*S. mahagoni* [24,45], *S. macrophylla* [1,32,33]
**85**	swietemahonin G	R_1_ = Tig, R_2_ = OH, R_3_ = OH	*S. mahagoni* [24,30,31,45,51], *S. macrophylla* [1]
**86**	swietemahonlide	R_1_ = Tig, R_2_ = H, R_3_ = H	*S. mahagoni* [24,45]
**87**	xylocarpin	R_1_ = AC, R_2_ = H, R_3_ = H	*S. mahagoni* [45], *S. macrophylla* [49]
**88**	humilin B	R_1_ = iBu, R_2_ = OH, R_3_ = H	*S. humilis* [38], *S. mahagoni* [41,45], *S. macrophylla* [49,58]
**89**	humilinolide A(methyl 3β-isobutyryloxy-2,6-dihydroxy-8α,30α-epoxy-l-oxo-meliacate)	R_1_ = iBu, R_2_ = OH, R_3_ = OH	*S. humilis* [39,40,41,61], *S. macrophylla* [58]
**90**	humilinolide B	R_1_ = iBu, R_2_ = OH, R_3_ = OAc	*S. humilis* [39,40,41]
**91**	humilinolide F	R_1_ = Tig, R_2_ = OAc, R_3_ = OAc	*S. humilis* [41],*S. macrophylla* [55]
**92**	6-deoxyswietemahonin A	R_1_ = COEt, R_2_ = H, R_3_ = H	*S. macrophylla* [48]
**93**	swielimonoid C	R_1_ = Piv, R_2_ = H, R_3_ = OH	*S. macrophylla* [60]
**94**	methyl 3*β*-acetoxy-2,6-dihydroxy-8*α*,30*α*-epoxy-l-oxo-meliacate	R_1_ = Ac, R_2_ = OH, R_3_ = OH	*S. macrophylla* [58]
**95**	methyl 3*β*-tigloyloxy-2-hvdroxy-8*α*,30*α*-epoxy-l-oxo-meliacate	R_1_ = Tig, R_2_ = OH, R_3_ = H	*S. macrophylla* [14,58] *S. mahagoni* [62]
**96**	6-*O*-acetylswietemahonin G	R_1_ = Tig, R_2_ = OH, R_3_ = OAc	*S. macrophylla* [14], *S. mahagoni* [62]
**97**	2-acetoxyswietemahonlide (swietemacrophin)	R_1_ = Tig, R_2_ = OAc, R_3_ = H	*S. macrophylla* [55]
**98**	humilinolide H	R_1_ = iBu, R_2_ = OAc, R_3_ = H	*S. humilis* [42]

**Table 4:** Structures and sources of phragmalin-type limonoids **99**–**153**.

No.	Compounds	Substitution Groups	Sources
**99**	swietenitin A	R_1_ = A_1_, R_2_ = Ac, R_3_ = Ac	*S. macrophylla* [63]
**100**	swietenitin B	R_1_ = A_2_, R_2_ = Ac, R_3_ = Ac	*S. macrophylla* [63]
**101**	swietenitin C	R_1_ = A_1_, R_2_ = Ac, R_3_ = COEt	*S. macrophylla* [63]
**102**	swietenitin D	R_1_ = A_1_, R_2_ = H, R_3_ = COEt	*S. macrophylla* [63]
**103**	swietenitin E	R_1_ = Tig, R_2_ = Ac, R_3_ = COEt	*S. macrophylla* [63]
**104**	swietenitin F	R_1_ = Tig, R_2_ = H, R_3_ = iBu	*S. macrophylla* [63]
**105**	swietenialide D	R_1_ = A_1_, R_2_ = H, R_3_ = COEt, R_4_ = OH	*S. mahagoni* [36]
**106**	swietenitin G	R_1_ = A_1_, R_2_ = Ac, R_3_ = Ac, R_4_ = OH	*S. macrophylla* [63]
**107**	swietenitin H	R_1_ = Tig, R_2_ = Ac, R_3_ = COEt, R_4_ = OAc	*S. macrophylla* [63]
**108**	2,11-diacetoxyswietenialide D	R_1_ = A_1_, R_2_ = Ac, R_3_ = COEt, R_4_ = OAc	*S. macrophylla* [63]
**109**	11-deoxyswietenialide D	R_1_ = A_1_, R_2_ = H, R_3_ = COEt, R_4_ = H	*S. macrophylla* [63]
**110**	2-acetoxyswietenialide D	R_1_ = A_1_, R_2_ = Ac, R_3_ = COEt, R_4_ = OH	*S. macrophylla* [63]
**111**	swietenialide A	R_1_ = Tig, R_2_ = Me, R_3_ = OMe, R_4_ = H, R_5_ = OH	*S. mahagoni* [36]
**112**	swietenialide B	R_1_ = Tig, R_2_ = Et, R_3_ = OMe, R_4_ = H, R_5_ = OH	*S. mahagoni* [36]
**113**	swietenialide C	R_1_ = A_1_, R_2_ = Me, R_3_ = OMe, R_4_ = H, R_5_ = OH	*S. mahagoni* [36]
**114**	swietenitin I	R_1_ = A_1_, R_2_ = Et, R_3_ = OMe, R_4_ = H, R_5_ = OH	*S. macrophylla* [63]
**115**	swietenitin J	R_1_ = A_1_, R_2_ = Et, R_3_ = OMe, R_4_ = Ac, R_5_ = OH	*S. macrophylla* [63]
**116**	swietenitin K	R_1_ = Tig, R_2_ = Et, R_3_ = OMe, R_4_ = Ac, R_5_ = OH	*S. macrophylla* [63]
**117**	swielimonoid D	R_1_ = A_1_, R_2_ = *α*-Et, R_3_ = *β*-OMe, R4 = Ac, R_5_ = OAc	*S. macrophylla* [60]
**118**	swielimonoid E	R_1_ = A_1_, R_2_ = *β*-Et, R_3_ = *α*-OMe, R_4_ = Ac, R_5_ = OAc	*S. macrophylla* [60]
**119**	swielimonoid F	R_1_ = A_1_, R_2_ = *β*-Et, R_3_ = *α*-OMe, R_4_ = H, R_5_ = OAc	*S. macrophylla* [60]
**120**	swielimonoid G	R_1_ = A_1_, R_2_ = *β*-Me, R_3_ = *α*-OMe, R_4_ = Ac, R_5_ = OAc	*S. macrophylla* [60]
**121**	swietenitin L	R_1_ = A_1_, R_2_ = H	*S. macrophylla* [63]
**122**	swietenitin M	R_1_ = A_1_, R_2_ = Ac	*S. macrophylla* [63]
**123**	swietenitin N	R_1_ = A_2_, R_2_ = COEt	*S. macrophylla* [64]
**124**	swietenitin O	R_1_ = A_2_, R_2_ = Ac	*S. macrophylla* [64]
**125**	swietenitin P	R_1_ = Tig, R_2_ = COEt	*S. macrophylla* [64]
**126**	epoxyfebrinin B	R_1_ = A_1_, R_2_ = Ac	*S. macrophylla* [64]
**127**	swietenitin Q		*S. macrophylla* [64]
**128**	swietenitin R	R_1_ = A_1_, R_2_ = H, R_3_ = COEt	*S. macrophylla* [64]
**129**	swietenitin S	R_1_ = Tig, R_2_ = Ac, R_3_ = COEt	*S. macrophylla* [64]
**130**	swietenitin T	R_1_ = A_1_, R_2_ = H, R_3_ = COEt	*S. macrophylla* [64]
**131**	swietenitin U	R_1_ = Tig, R_2_ = H, R_3_ = Ac	*S. macrophylla* [64]
**132**	swietenitin V		*S. macrophylla* [64]
**133**	swietenitin W	R = H	*S. macrophylla* [64]
**134**	swietenitin X	R = Me	*S. macrophylla* [64]
**135**	swietephragmin A	R_1_ = Tig, R_2_ = OAc, R_3_ = H, R_4_ = iPr, R_5_ = H	*S. mahagoni* [30]
**136**	swietephragmin B	R_1_ = Tig, R_2_ = OAc, R_3_ = H, R_4_ = A_3_, R_5_ = H	*S. mahagoni* [30]
**137**	swietephragmin C	R_1_ = Tig, R_2_ = OH, R_3_ = H, R_4_ = A_3_, R_5_ = H	*S. mahagoni* [30]
**138**	swietephragmin D	R_1_ = Tig, R_2_ = OH, R_3_ = H, R_4_ = iPr, R_5_ = H	*S. mahagoni* [30]
**139**	swietephragmin E	R_1_ = Tig, R_2_ = OH, R_3_ = OH, R_4_ = A_3_, R_5_ = H	*S. mahagoni* [30]
**140**	swietephragmin F	R_1_ = Tig, R_2_ = OH, R_3_ = H, R_4_ = Et, R_5_ = H	*S. mahagoni* [30]
**141**	swietephragmin G	R_1_ = Tig, R_2_ = OH, R_3_ = H, R_4_ = Me, R_5_ = H	*S. mahagoni* [30]
**142**	6-*O*-acetylswietephragmin E	R_1_ = Tig, R_2_ = OH, R_3_ = OAc, R_4_ = A_3_, R_5_ = H	*S. macrophylla* [66]
**143**	12*α*-acetoxyswietephragmin C	R_1_ = Tig, R_2_ = OH, R_3_ = H, R_4_ = A_3_, R_5_ = OAc	*S. macrophylla* [66]
**144**	3*β*-*O*-destigloyl-3*β*-*O*-benzoyl-6-*O*-acetylswietephragmin E	R_1_ = Bz, R_2_ = OH, R_3_ = OAc, R_4_ = A_3_, R_5_ = H	*S. macrophylla* [66]
**145**	3*β*-*O*-destigloyl-3*β*-*O*-benzoyl-12*α*-acetoxyswietephragmin C	R_1_ = Bz, R_2_ = OH, R_3_ = H, R_4_ = A_3_, R_5_ = OAc	*S. macrophylla* [66]
**146**	12*α*-acetoxyswietephragmin D	R_1_ = Tig, R_2_ = OH, R_3_ = H, R_4_ = iPr, R_5_ = OAc	*S. macrophylla* [66]
**147**	3*β*-*O*-destigloyl-3*β*-*O*-benzoyl-12*α*-acetoxyswietephragmin D	R_1_ = Bz, R_2_ = OH, R_3_ = H, R_4_ = iPr, R_5_ = OAc	*S. macrophylla* [66]
**148**	6-*O*-acetyl-3′-demethylswietephragmin E	R_1_ = Tig, R_2_ = OH, R_3_ = OAc, R_4_ = iPr, R_5_ = H	*S. macrophylla* [66]
**149**	swietephragmin H	R_1_ = Tig, R_2_ = OAc, R_3_ = H, R_4_ = Et, R_5_ = H	*S. macrophylla* [65]
**150**	swietephragmin I	R_1_ = Tig, R_2_ = OAc, R_3_ = H, R_4_ = Me, R_5_ = H	*S. macrophylla* [65]
**151**	swietephragmin J	R_1_ = Tig, R_2_ = OAc, R_3_ = H, R_4_ = Et, R_5_ = OH	*S. macrophylla* [65]
**152**	swietenialide E		*S. mahagoni* [57]
**153**	11-hydroxyswietephragmin B		*S. mahogani* [31]

**Table 5:** Structures and sources of polyoxyphragmalin-type limonoids **154**–**164**.

**No.**	**Compounds**	**Substitution Groups**	**Sources**
**154**	khayanolide E	R_1_ = O, R_2_ = Ac	*S. macrophylla* [37]
**155**	1-*O*-acetylkhayanolide B	R_1_ = *β*-OH, *α*-H, R_2_ = Ac	*S. macrophylla* [37]
**156**	1-*O*-deacetylkhayanolide E	R_1_ = O, R_2_ = H	*S. macrophylla* [37]
**157**	khayanolide B	R_1_ = *β*-OH, *α*-H, R_2_ = H	*S. macrophylla* [37]
**158**	khayalactone		*S. macrophylla* [37]
**159**	1-*O*-acetylkhayanolide A	R = Ac	*S. macrophylla* [37]
**160**	khayanolide A	R = H	*S. macrophylla* [37]
**161**	swietemahalactone		*S. mahagoni* [67]
**162**	swiemahogin B		*S. mahagoni* [34]
**163**	swietenine J	R_1_ = Ac, R_2_ = H, R_3_ = H, R_4_ = H	*S. macrophylla* [37]
**164**	swietemacrophine	R_1_ = Tig, R_2_ = OTig, R_3_ = OH, R_4_ = OAc	*S. macrophylla* [65]

**Table 6:** Antifeedant effects of limonoids.

Compounds	Insect and Antifeedant Activity
swietenolide (**23**)	*Spodoptera frugiperda* AI = 94.1 ± 2.90 (1000 ppm) [1], DC_50_ = 80.6 ± 1.1 (mg/L) [68]
6-acetylswietenolide (**25**)	*S. frugiperda* AI = 72.2 ± 19.60 (1000 ppm) [1]
3,6-*O*,*O*-diacetylswietenolide (**27**)	*S. frugiperda* AI = 72.0 ± 9.38 (1000 ppm) [1]
swietemahonin F (**84**)	*S. frugiperda* AI = 70.2 ± 8.90 (1000 ppm) [1]
swietenine (42)	*S. frugiperda* DC_50_ = 2.49 ± 1.44 (mg/L) [68]
2-hydroxyswietenine (**55**)	*S. frugiperda* DC_50_ = 65.8 ± 1.2 (mg/L) [68]
swietemahonin G (**85**)	*S. frugiperda* DC_50_ = 13.8 ± 1.2 (mg/L) [68], *Spodoptera littoralis*, MAC values = 300 [31]
3,6-*O*,*O*-diacetylswietenolide (**27**)	*S. frugiperda,* DC_50_ = 4.65 ± 1.33 (mg/L) [68]
6-*O*-acetylswietemahonin G (**96**)	*S. littoralis*, MAC values = 500 [62]
swietenialides A–E (**111–113**, **117**, **118**)	*S. littoralis*, MAC values = 1000 [36]
7-deacetoxy-7-oxogedunin (**5**)	*S. littoralis*, MAC values = 1000 [31]
methyl 6-hydroxyangolensate (**16**)	*S. littoralis*, MAC values = 500 [31]
6-*O*-acetyl-2-hydroxyswietenin (**52**)	*S. littoralis*, MAC values = 500 [31]
2-hydroxy-6-deacetoxyswietenine (**54**)	*S. littoralis*, MAC values = 500 [31]
2-hydroxyswietenine (**55**)	*S. littoralis*, MAC values = 500 [31]
swietephragmin H (**149**)	*S. littoralis*, MAC values = 1000 [31]
swietephragmin I (**150**)	*S. littoralis*, MAC values = 500 [31]
11-hydroxyswietephragmin B (**153**)	*S. littoralis*, MAC values = 1000 [31]
humilinolide B (**90**)	*Sitophilus oryzae,* AI = 79.7 ± 16.7 [69]
humilinolide C (**50**)	*S. oryzae,* AI = 24.8 ± 1.0 [69]
humilinolide D (**51**)	*S. oryzae,*AI = 65.2 ± 11.1 [69]
